# Evaluation of Machine Learning Approaches for Predicting Warfarin Discharge Dose in Cardiac Surgery Patients: Retrospective Algorithm Development and Validation Study

**DOI:** 10.2196/47262

**Published:** 2023-12-06

**Authors:** Lindsay Dryden, Jacquelin Song, Teresa J Valenzano, Zhen Yang, Meggie Debnath, Rebecca Lin, Jane Topolovec-Vranic, Muhammad Mamdani, Tony Antoniou

**Affiliations:** 1 Unity Health Toronto Toronto, ON Canada

**Keywords:** algorithm, anticlotting, anticoagulant, anticoagulation, blood thinner, cardiac, cardiology, develop, dosage, international normalized ratio, machine learning, medical informatics, pharmacology, postoperative, predict, prescribe, prescription, surgery, surgical, validate, validation, warfarin administration and dosage, warfarin

## Abstract

**Background:**

Warfarin dosing in cardiac surgery patients is complicated by a heightened sensitivity to the drug, predisposing patients to adverse events. Predictive algorithms are therefore needed to guide warfarin dosing in cardiac surgery patients.

**Objective:**

This study aimed to develop and validate an algorithm for predicting the warfarin dose needed to attain a therapeutic international normalized ratio (INR) at the time of discharge in cardiac surgery patients.

**Methods:**

We abstracted variables influencing warfarin dosage from the records of 1031 encounters initiating warfarin between April 1, 2011, and November 29, 2019, at St Michael’s Hospital in Toronto, Ontario, Canada. We compared the performance of penalized linear regression, k-nearest neighbors, random forest regression, gradient boosting, multivariate adaptive regression splines, and an ensemble model combining the predictions of the 5 regression models. We developed and validated separate models for predicting the warfarin dose required for achieving a discharge INR of 2.0-3.0 in patients undergoing all forms of cardiac surgery except mechanical mitral valve replacement and a discharge INR of 2.5-3.5 in patients receiving a mechanical mitral valve replacement. For the former, we selected 80% of encounters (n=780) who had initiated warfarin during their hospital admission and had achieved a target INR of 2.0-3.0 at the time of discharge as the training cohort. Following 10-fold cross-validation, model accuracy was evaluated in a test cohort comprised solely of cardiac surgery patients. For patients requiring a target INR of 2.5-3.5 (n=165), we used leave-p-out cross-validation (p=3 observations) to estimate model performance. For each approach, we determined the mean absolute error (MAE) and the proportion of predictions within 20% of the true warfarin dose. We retrospectively evaluated the best-performing algorithm in clinical practice by comparing the proportion of cardiovascular surgery patients discharged with a therapeutic INR before (April 2011 and July 2019) and following (September 2021 and May 2, 2022) its implementation in routine care.

**Results:**

Random forest regression was the best-performing model for patients with a target INR of 2.0-3.0, an MAE of 1.13 mg, and 39.5% of predictions of falling within 20% of the actual therapeutic discharge dose. For patients with a target INR of 2.5-3.5, the ensemble model performed best, with an MAE of 1.11 mg and 43.6% of predictions being within 20% of the actual therapeutic discharge dose. The proportion of cardiovascular surgery patients discharged with a therapeutic INR before and following implementation of these algorithms in clinical practice was 47.5% (305/641) and 61.1% (11/18), respectively.

**Conclusions:**

Machine learning algorithms based on routinely available clinical data can help guide initial warfarin dosing in cardiac surgery patients and optimize the postsurgical anticoagulation of these patients.

## Introduction

Warfarin is a commonly prescribed oral anticoagulant for patients who have undergone cardiac surgery and remains the only guideline-endorsed anticoagulant for patients with mechanical heart valves. However, considerable interindividual variability exists in the response to warfarin, with patient age, BMI, concomitant medications, and genetic status imparting considerable influence on warfarin maintenance dose requirements [1-5]. Complicating matters in cardiac surgery patients is an exaggerated sensitivity to the effects of warfarin in the immediate postoperative period, predisposing individuals to supratherapeutic international normalized ratio (INR) values and bleeding [3,6,7]. Consequently, close monitoring and frequent dosage adjustments are needed to optimize warfarin therapy and prevent thromboembolic events and bleeding in these patients [8].

Warfarin dosing prediction algorithms are commonly used by clinicians to optimize treatment and reduce the unpredictability of warfarin responses [9,10]. Recently, machine learning methods have been used for developing and validating algorithms that leverage patient information to guide warfarin dosing and facilitate individualized treatment [11-16]. A systematic review of 266 studies describing 433 warfarin dosing algorithms found that most algorithms were derived using both clinical and genetic variables (344/433, 79.4%), emphasized dose initiation (373/433, 86.1%) rather than discharge or maintenance doses, and presented a regression formula that could be used to compute a weekly or daily dose (239/433, 55.2%) [17]. The most commonly reported outcomes were the mean absolute and mean prediction errors, with few algorithms undergoing external validation or clinical utility assessment to gauge their performance in a clinical setting relative to clinicians. Moreover, the majority (280/433, 64.7%) of algorithms were developed using linear regression and may therefore not accurately characterize the complex relationships between warfarin dosing and patient features [17].

Considering the complex relationship between warfarin and patient characteristics, predictive dosing algorithms have been developed using machine learning methods that can more readily accommodate nonlinear relationships and interactions between features. A recent systematic review summarized the characteristics and quality of 23 studies investigating nonlinear machine learning algorithms for warfarin dose prediction [13]. Most (78%) of the studies were conducted in Asia or at sites associated with the International Warfarin Pharmacogenetics Consortium, with China being the most represented single country among the included studies (9/23, 39%). The most common demographic and clinical predictors were age (21/23, 91%), weight (17/23, 74%), height (12/23, 52%), and concomitant administration of amiodarone (11/23, 48%), while CYP2C9 (14/23, 61%), VKORC1 (14/23, 61%), and CYP4F2 (5/23, 22%) were the most common genetic predictors. The most reported outcome measures were the mean absolute error (MAE) and whether the predicted dose was within 20% of the actual dose, derived in 91% (21/23) and 83% (19/23) of studies, respectively. The majority (16/23, 70%) of studies focused on model development with internal validation only. In addition, the studies were found to be at high risk of bias, with poor handling of missing data (20/23, 87%) and a small sample size (15/23, 65%) being the factors most commonly contributing to bias.

Recent studies have evaluated more advanced methods for guiding warfarin dosing in cardiac surgery patients. Specifically, a study of 13,639 eligible patients identified in the Chinese Low Intensity Anticoagulant Therapy after Heart Valve Replacement database compared the performance of a previously described 3-layer Back Propagation Neural Network (BPNN) model with multiple linear regression for predicting the warfarin maintenance dose in patients undergoing heart valve replacement [14,15]. Results demonstrated a slightly improved performance for the BPNN model, with a MAE in the external validation group of 0.740 (95% CI 0.671-0.810) compared with 0.750 (95% CI 0.673-0.814) with multiple linear regression [14]. The percentage of patients in the external validation group whose predicted absolute error between the predicted and actual doses was within 20% of the actual dose was also slightly better with the BPNN algorithm (59.7%) compared with multiple linear regression (56.6%).

A recent study has also examined the potential for reinforcement learning in predicting the daily warfarin dose required for patients undergoing surgical valve replacement [18]. The study was conducted in China and involved 10,408 patients, partitioned into training (n=8216), validation (n=932), and test (n=1260) data sets. The primary outcome was the proportion of patients in the test data set categorized as excellent responders, defined as the absence of an INR >3.0 during the entire postoperative stay and having a discharge INR within the target range of 1.8-2.5. The individual components of the primary outcome, defined as the proportion of test patients who were safety responders (ie, no INRs >3.0 after warfarin initiation) and target responders (ie, discharge INR within 1.8-2.5), were examined in secondary analyses. Comparisons were made between the reinforcement learning algorithm, clinician practice, and other machine learning algorithms. Overall, the reinforcement learning algorithm outperformed clinician practice with respect to the proportion of the test set patients being excellent responders (80.8% vs 41.6%; *P*<.001). For the secondary outcomes, the safety responder ratio (RR; 83.1% vs 99.5%; RR 0.83, 95% CI 0.81-0.86; *P*<.001) and the target responder ratio (49.7% vs 81.1%; RR 0.61, 95% CI 0.58-0.65; *P*<.001) were lower for clinicians relative to the reinforcement learning algorithm. Similarly, performance for all outcomes was significantly better with the reinforcement learning algorithm relative to other machine learning approaches. This study therefore demonstrated the potential for applying reinforcement learning to improve outcomes in patients undergoing cardiac surgery who are treated with warfarin. However, the generalizability of these findings to other settings may be limited by the generally lower anticoagulation intensity examined in this study relative to clinical practice in Western countries.

Our center, St Michael’s Hospital, is a large, inner-city academic hospital in Toronto, Ontario, Canada, that performs over 1000 cardiac surgeries per year. Pharmacists and clinicians caring for cardiac surgery patients identified challenges discharging warfarin-treated patients with a therapeutic INR as a problem potentially amenable to machine learning-based solutions for the hospital health care analytics team. However, generalizing the findings from existing literature to our setting and patient population was considered infeasible and inappropriate for several reasons. First, St Michael’s Hospital treats a diverse inner-city population that was not reflected in the studies conducted to date, with most algorithms developed and validated in predominantly Asian and White populations [13,17]. Second, the clinical utility of many algorithms, particularly in cardiac surgery patients, has not been evaluated. It is therefore unclear how these algorithms compare to existing clinical practice [13]. Third, many algorithms rely on the use of pharmacogenetic information that is not readily available in most clinical settings, including ours [16,19]. In addition, although neural networks and reinforcement learning are promising approaches for predicting warfarin dose in cardiac surgery patients, the large sample sizes required for their derivation and validation were prohibitive at our site. There was therefore a need to develop an algorithm using routinely available patient data that respected our data limitations and allowed for more complex relationships between patient variables and warfarin dose. Considering these needs, we developed and validated algorithms for predicting the warfarin dose required to attain a therapeutic INR at the time of discharge among patients who have undergone cardiac surgery using commonly available clinical and demographic data.

## Methods

### Setting

We conducted a retrospective algorithm development and validation study using the data of all patients initiating warfarin during a hospital admission at St Michael’s Hospital between April 1, 2011, and November 29, 2019. Warfarin dosing in cardiac surgery patients at St Michael’s Hospital is led primarily by team pharmacists under a medical directive. The study was undertaken as a collaboration between clinical staff and the data scientists of the Data Science and Advanced Analytics team, a service-based–health care analytics group at St Michael’s Hospital with expertise in machine learning.

### Data Sources

We used the St Michael’s Hospital Enterprise Data Warehouse (EDW), which integrates and stores structured and unstructured data at the patient level from several hospital electronic databases, including inpatient and outpatient electronic medical charts, inpatient pharmacy records, and results of laboratory and medical imaging investigations [20]. The EDW is updated daily using automated algorithms that abstract, clean, and link data from separate hospital sources using a patient’s unique medical record number.

### Study Population and Outcomes

Our base population included all patients newly initiating warfarin during the course of hospital admission between April 1, 2011, and November 29, 2019, regardless of indication. From within this cohort, we included individuals administered warfarin and undergoing INR testing on at least three separate occasions during a 7-day period. Next, we excluded individuals admitted for palliative care and those who died during their admission, patients transferring to and from the mental health inpatient unit during their admission, and individuals with discharge dispositions undermining outcome ascertainment, including leaving against medical advice and not returning to the hospital following an authorized pass. Finally, we retained only those individuals discharged with a therapeutic INR, defined in this study as ranging from 2.5 to 3.5 for patients receiving mechanical heart valves and from 2.0 to 3.0 for all other patients [21].

### Outcome Measures and Features

Our primary outcome was the warfarin dose needed to attain a therapeutic INR at the time of discharge following cardiovascular surgery. The specific procedures included mechanical mitral valve surgery (target INR 2.5-3.5), mechanic aortic valve surgery (target INR 2.0-3.0), prosthetic or tissue mitral or tricuspid valve surgery (target INR 2.0-3.0), and new-onset atrial fibrillation of more than 48 hours duration following aortic surgery or coronary bypass surgery (target INR 2.0-3.0), with target INRs based on clinical practice guidelines [22-24]. We identified demographic and clinical determinants of the final warfarin dose from past research and consultation with experts and included those variables that could be abstracted from patient records as features in our predictive models (Textbox 1). To account for heterogeneity in warfarin sensitivity and dosing requirements, we also included the change in INR and warfarin dose from their immediately preceding values, as well as multiplications of the warfarin dose and its subsequent INR measurement, as model features.

**Model features and descriptions.**

**Model features and descriptions:**

- Warfarin 1: First postoperative warfarin dose (mg/day)
- Warfarin 2: Second postoperative warfarin dose (mg/day)
- Warfarin 3: Third postoperative warfarin dose (mg/day)
- Warfarin difference 1: The absolute difference between the 1st and 2nd warfarin doses
- Warfarin difference 2: The absolute difference between the 2nd and 3rd warfarin doses
- Warfarin difference 3: The absolute difference between the 1st and 3rd warfarin doses
- International normalized ratio (INR) 1: First postoperative INR measurement following warfarin administration.
- INR 2: Second postoperative INR measurement following warfarin administration.
- INR 3: Third postoperative INR measurement following warfarin administration.
- INR difference 1: The absolute difference between the 1st and 2nd INR measurements.
- INR difference 2: The absolute difference between the 2nd and 3rd INR measurements.
- INR difference 3: The absolute difference between the 1st and 3rd INR measurements.
- Times 1: The multiplication of Warfarin 1 and INR 1
- Times 2: The multiplication of Warfarin 2 and INR 2
- Times 3: The multiplication of Warfarin 3 and INR 3
- Sex: Patient’s sex.
- Age: Patient’s age at time of admission.
- Indication: Binary indicators for one of the following procedure types that the patient received: cardiac valve replacement, cardiac valve replacement except percutaneous transluminal approach, coronary artery bypass graft, and others.
- Comorbidities: Binary indicators for the presence of any of the following comorbidities: myocardial infarction (MI), peripheral vascular disorders (PVD), congestive heart failure (CHF), rheumatic heart disease, diabetes, renal disease, peptic ulcer disease (PUD), HIV, and stroke.
- Medication groups: Binary indicators for the presence of any of the following medication groups: aspirin, clopidogrel, ticagrelor, amiodarone, fibrates, nonsteroidal anti-inflammatory drugs (NSAIDs), selective serotonin reuptake inhibitor (SSRIs), phenytoin, carbamazepine, fluconazole, fluoroquinolones, metronidazole, rifampin, and trimethoprim-sulfamethoxazole.

### Statistical Models

We compared the performance of 5 different models to predict the warfarin dose required to attain discharge INRs of 2.0-3.0 and 2.5-3.5. Specifically, we examined penalized linear regression, k-nearest neighbors (kNN), random forest regression, gradient boosting, and multivariate adaptive regression splines (MARS). Penalized linear regression is an extension of ordinary least squares that includes a regularization constraint in the model to shrink coefficient values toward zero relative to the least squares estimates [25,26]. Random forest regression is an ensemble of independent decision trees created by using random bootstrap samples of the training observations and random subsets of the candidate variables in each split of the tree [27,28]. The final prediction is obtained by calculating the mean of the predictions from the individual trees comprising the forest. In contrast to random forest, which is an ensemble of independent trees, gradient boosting involves the stepwise construction of many small regression trees from the pseudoresiduals of previous trees [25,29,30]. MARS is a nonparametric modeling method that avoids the questionable linearity assumption of regular linear regression. Specifically, MARS approximates the nonlinear relationship between response and predictor variables by fitting the data into piecewise linear regression functions [31]. kNN regression is a nonparametric algorithm that makes predictions by averaging the outcomes of the observations most similar to the target, weighted by the inverse of their distance [25]. We also determined the performance of an ensemble model combining the predictions of the 5 regression models. For each model, we used grid search to tune hyperparameters, trying multiple parameter settings over a predefined range of values and selecting the values of each hyperparameter providing the best prediction (Table S1 in Multimedia Appendix 1). We also used cross-validation to estimate model performance and generalizability with unseen data. In this study, we used 2 distinct cross-validation strategies tailored to the characteristics of our patient groups. For patients within the target INR range of 2.0-3.0, we implemented a 10-fold cross-validation approach. This method divides the data into 10 subsets, or “folds,” and iteratively uses 9 folds for training and 1 fold for validation, ensuring comprehensive model assessment while mitigating overfitting by repeating this process 10 times with different validation sets. This approach was selected for its balance between robustness and computational efficiency. Conversely, for patients with a target INR of 2.5-3.5, who were characterized by a notably limited data set, we adopted leave-p-out cross-validation with p=3 to ensure even splits of the 165 observations. Leave-p-out cross-validation is particularly advantageous in scenarios with limited data, as it systematically excludes p data points at a time, creating multiple validation sets. This approach was crucial because the small size of the data set made it impractical to create a separate testing set, thus allowing us to make the most of the available data while achieving reliable results.

We quantified model performance using the MAE, defined as the absolute value of the difference between the true and predicted doses of warfarin required to attain the required discharge INR. A predicted dose within 1 mg of the predicted dose is considered a reasonable measure of predictive ability [32]. We also determined the proportion of predictions within 20% of the true warfarin dose. We selected these measures because they are commonly used in studies of warfarin dosing algorithms, thereby allowing us to facilitate a comparison with past research [13,17,33,34]. In addition, we compared our findings with clinical algorithms developed by Gage and colleagues [33] and the International Warfarin Pharmacogenetics Consortium [34]. We specifically chose these algorithms because they are among the most externally validated and clinically assessed algorithms. Moreover, the Gage algorithm has been operationalized as a freely available web-based calculator [35] that is commonly used for estimating warfarin doses in various clinical settings, including heart valve replacement. Because of the skewed distribution of warfarin doses, we replicated our analyses using a logarithmic transformation of the warfarin dose and compared these findings with those generated by models predicting the untransformed dose.

### Model Creation

Because the intensity of postsurgical anticoagulation required for a given patient is determined by the nature of cardiac surgery performed, we developed and validated separate models for predicting the warfarin dose required for achieving a discharge INR of 2.0-3.0 in patients undergoing all forms of cardiac surgery other than mechanical mitral valve replacement (ie, prosthetic tissue valve replacement, postsurgical atrial fibrillation, and mechanical aortic valve replacement) and a discharge INR of 2.5-3.5 in patients receiving a mechanical mitral valve replacement.

We selected 80% of encounters (n=780) who had initiated warfarin during their hospital admission and had achieved a target INR of 2.0-3.0 at the time of discharge as the training cohort. Following 10-fold cross-validation, model accuracy was evaluated in the test cohort, comprising the remaining 20% (n=195) of patients. To ensure we had an adequate number of patients for model training, we included patients initiating warfarin for reasons unrelated to cardiac surgery (eg, thromboembolic disease) in the training cohort. This approach is similar to that of past studies developing warfarin dosing algorithms for a specific target INR, with subsequent dosing tools based on these algorithms incorporating clinical indication as one of several variables for predicting a warfarin dose [13,17,33,34]. However, because our primary motivation was to determine a tool that could guide warfarin dosing specifically in cardiac surgery patients, our test cohort consisted solely of cardiac surgery patients, thereby ensuring that the performance of the final model would be determined with the target study population. The cardiac surgery patient cohort was defined as any patient whose service was cardiovascular surgery or patients with the following procedures: cardiac valve replacement, cardiac valve repair, or coronary artery bypass graft (Table 1). The model with the lowest MAE in the test cohort was selected as the final model for predicting the stable warfarin dose needed to attain a discharge INR of 2.0-3.0.

**Figure 1:**
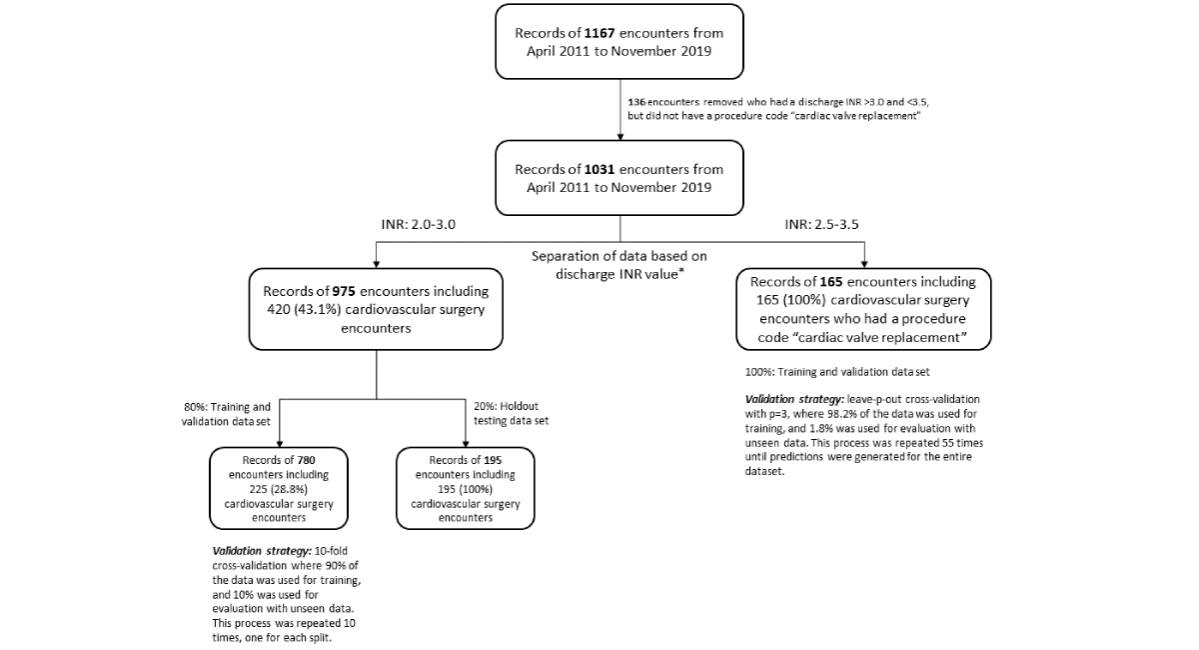
Model development study design outlining cohort, data splits, and model validation strategies. INR: international normalized ratio. *There are 109 encounters who are represented in both INR groups of 2.0-3.0 and 2.5-3.5. These were cardiovascular surgery encounters who had a procedure code "cardiac valve replacement" and had a discharge INR value between 2.5-3.0.

Because cardiac surgery patients requiring a target INR of 2.5-3.5 represent those individuals requiring a mechanical mitral valve replacement, we did not include noncardiac surgery patients in this data set, resulting in a smaller sample of 165 patients for model training and validation. These 165 patients all had cardiac valve replacements. Consequently, rather than partitioning the data into 2 separate training and test sets, we used leave-p-out cross-validation, where p=3 observations, to estimate model performance. Figure 1 outlines the study cohort, data splits, and validation strategies.

**Table 1:** Characteristics of encounters initiating warfarin while hospitalized between April 1, 2011, and November 29, 2019.

Characteristics		INR^a^ 2.0-3.0				INR 2.5-3.5
		Training and validation (n=780)		Testing (n=195)	Training and validation (n=165)	
Age (years), median (IQR)		67 (56-78)		67 (58.5-76)	65 (55-75)	
Sex (male), n (%)		466 (59.7)		125 (64.1)	98 (59.4)	
**Procedure, n (%)**						
	Cardiac valve replacement		156 (20)	122 (62.6)	165 (100)	
	Cardiac valve repair^b^		21 (2.7)	18 (9.2)	0 (0)	
	Coronary artery bypass graft (new onset atrial fibrillation)		41 (5.3)	40 (20.5)	0 (0)	
	Other^c^		562 (72.1)	15 (7.7)	0 (0)	
**Service^d^, n (%)**						
	Cardiovascular surgery	120 (15.4)		195 (100)	110 (66.7)	
	Cardiology	144 (18.5)		0 (0)	32 (19.4)	
	Intensive coronary care	59 (7.6)		0 (0)	12 (7.3)	
	Intensive care cardiovascular	16 (2.1)		0 (0)	10 (<6.1)	
	Other^e^	441 (56.5)		0 (0)	10 (<6.1)	
**Potentially interacting medications, n (%)**						
	Amiodarone	230 (29.5)		121 (62.1)	105 (63.6)	
	Fluoroquinolones	164 (21)		35 (17.9)	26 (15.8)	
	Clopidogrel	97 (12.4)		14 (7.2)	16 (9.7)	
	Trimethoprim/sulfamethoxazole	52 (6.7)		6 (3.1)	5 (3)	
	Phenytoin	49 (6.3)		13 (6.7)	6 (3.6)	
	Metronidazole	47 (6)		<5 (<2.6)	<5 (<3)	
	Fluconazole	16 (2.1)		<5 (<2.6)	<5 (<3)	
	Ticagrelor	15 (1.5)		0 (0)	<5 (<3)	
	Fibrates	7 (0.9)		<5 (<2.6)	<5 (<3)	
	Rifampin	6 (0.8)		<5 (<2.6)	0 (0)	
	Carbamazepine	<5 (<0.6)		0 (0)	0 (0)	
	Aspirin	<5 (<0.6)		<5 (<2.6)	<5 (<3)	
**Comorbidities, n (%)**						
	Congestive heart failure	310 (39.7)		59 (30.3)	62 (37.6)	
	Stroke	123 (15.8)		16 (8.2)	17 (10.3)	
	Myocardial infarction	121 (15.5)		22 (11.3)	15 (9.1)	
	Peripheral vascular disorders	95 (12.2)		21 (10.8)	26 (15.8)	
	Renal disease	134 (17.2)		16 (8.2)	15 (9.1)	
	Rheumatic heart disease	17 (2.2)		<5 (<2.6)	<5 (<3)	
	Peptic ulcer disease	25 (3.2)		0 (0)	0 (0)	
	HIV	11 (1.4)		<5 (<2.6)	<5 (<3)	
	Diabetes	261 (33.5)		50 (25.6)	46 (27.9)	
**Warfarin doses (mg/day), median (IQR)**						
	First dose	5 (3-5)		3 (2.5-5)	3 (2.5-5)	
	Second dose	5 (3-5)		3.5 (2.75-5)	4 (2.5-5)	
	Third dose	5 (3-5)		3 (2-5)	4 (2.5-5)	
	Final warfarin dose^f^	3.5 (2.5-5)		3 (2-5)	3 (2.5-4)	
**INR measurements, median (IQR)**						
	INR after first Warfarin dose	1.31 (1.18-1.60)		1.3 (1.19-1.52)	1.35 (1.22-1.52)	
	INR after second Warfarin dose	1.57 (1.34-2.09)		1.6 (1.375-2)	1.66 (1.345-2.09)	
	INR after third Warfarin dose	1.98 (1.59-2.48)		1.94 (1.62-2.45)	2.06 (1.69-2.6)	
	Discharge INR	2.41 (2.22-2.62)		2.42 (2.2-2.625)	2.85 (2.63-3.12)	

^a^INR: international normalized ratio.

^b^Excludes percutaneous transluminal approach.

^c^Procedures in the “Other” category are all other procedures and conditions not listed above, including but not limited to pulmonary embolism, heart failure without coronary angiogram, and ischemic events of the central nervous system.

^d^Patients who have had a cardiac surgery procedure may not always have “cardiovascular surgery” as their service.

^e^Services in the “Other” category are all other services not listed above, including orthopedics, intensive care medical, general medicine, vascular surgery, intensive care trauma, neurosurgery, general surgery acute care, and other hospital services with less than 2% of total patients.

^f^The dose required to achieve therapeutic INR at discharge following cardiac surgery.

### Retrospective Case Series

On September 6, 2021, the algorithm was deployed as a web-based dosing calculator for predicting the warfarin dose required to attain a therapeutic INR at the time of discharge among patients undergoing cardiovascular surgery. To preliminarily ascertain how the algorithm performed in clinical practice, we retrospectively determined the proportion of cardiovascular surgery patients discharged with a therapeutic INR before (April 2011 and July 2019) and following (September 2021 and May 2, 2022) the implementation of the web-based calculator. We also reviewed the charts of patients with discrepancies between the dose predicted by the algorithm and the actual discharge warfarin dose and patients who were not discharged with a therapeutic INR to identify whether specific patient features (eg, smoking history and BMI) not included in the development and validation of the algorithm could be undermining the performance of the web-based calculator in clinical practice.

### Ethical Considerations

This study was approved by the Research Ethics Board of Unity Health Toronto, Ontario, Canada (reference #16-371). Because fully anonymized data were used, the need for informed consent was waived by the Research Ethics Board.

## Results

### Algorithm Validation

We identified 1031 encounters initiating warfarin while hospitalized between April 1, 2011, and November 29, 2019, who met the inclusion criteria, of whom 334 (32.4%) received a cardiac valve replacement and 142 (13.8%) underwent other forms of cardiac surgery. The majority (628/1031, 60.9%) of patients were male, and the median age was 67 (IQR 56-77) years (Table 1).

In our analysis predicting the warfarin dose for a discharge INR of 2.0-3.0, random forest regression was the best performing model in the test set of cardiac surgery patients, with an MAE of 1.13 mg and 39.5% of predictions falling within 20% of the actual dose (Table 2, Figure S1 in Multimedia Appendix 1).

The MAEs for the penalized linear regression, MARS, gradient boosting, kNN regression, and ensemble models were 1.22 mg, 1.25 mg, 1.15 mg, 1.26 mg, and 1.16 mg, respectively, with the proportion of predicted doses falling within 20% of the actual dose ranging from 32.8% to 36.9% (Table 1). Logarithmic transformation of the warfarin dose did not result in a better-performing model than random forest regression of the untransformed dose (Table 2).

For predictions of the warfarin dose required for a discharge INR of 2.5-3.5, the ensemble model combining the results of the 5 regression models performed best among patients undergoing mechanical mitral valve replacement. Specifically, the MAE for the ensemble model was 1.11 mg, with 43.6% of predictions being within 20% of the actual dose. The mean average errors for the penalized linear regression, random forest regression, MARS, gradient boosting, and kNN regression models were 1.17 mg, 1.16 mg, 1.30 mg, 1.27 mg, and 1.26 mg, respectively, with 37.0%-41.2% of predictions being within 20% of the actual dose (Table 3, Figure S2 in Multimedia Appendix 1). Logarithmic transformation of the warfarin dose did not improve model performance.

**Table 2:** Model performance for predicting the warfarin dose for a discharge international normalized ratio (INR) of 2.0-3.0 using validation and testing data sets.

Model	Outcome transformation	Mean absolute error (MAE; mg), 95% CI		Proportion of predictions within 20% of true dose (%), 95% CI			Correlation coefficient, 95% CI	
		Validation	Test	Validation	Test	Validation		Test
Penalized regression	None	1.15 (1.07-1.23)	1.22 (1.08-1.37)	46.9 (43.5-50.4)	35.9 (28.7-42.6)	0.728 (0.688-0.772)		0.550 (0.466-0.629)
MARS^a^	None	1.20 (1.12-1.29)	1.25 (1.10-1.41)	42.6 (39.0-46.2)	33.3 (26.2-39.5)	0.720 (0.678-0.767)		0.548 (0.447-0.639)
kNN^b^	None	1.50 (1.41-1.59)	1.26 (1.09-1.41)	33.2 (29.7-36.5)	35.4 (28.2-42.0)	0.569 (0.521-0.621)		0.462 (0.353-0.573)
Random forest regression	None	1.17 (1.09-1.25)	1.13 (0.99-1.27)	44.5 (40.9-48.1)	39.5 (32.8-46.2)	0.728 (0.686-0.773)		0.579 (0.489-0.672)
Gradient boosting	None	1.25 (1.17-1.34)	1.15 (1.01-1.29)	41.7 (38.2-45.0)	36.9 (29.7-43.6)	0.690 (0.640-0.743)		0.596 (0.498-0.697)
Ensemble model	None	1.13 (1.04-1.21)	1.16 (1.02-1.29)	48.2 (44.5-51.5)	32.8 (26.2-39.5)	0.742 (0.701-0.786)		0.582 (0.494-0.665)
Penalized regression	Logarithm	1.18 (1.09-1.27)	1.21 (1.04-1.35)	43.7 (40.3-47.4)	34.4 (27.7-41.0)	0.716 (0.673-0.763)		0.535 (0.440-0.621)
MARS	Logarithm	1.14 (1.05-1.22)	1.18 (1.02-1.33)	46.3 (42.9-50.0)	34.9 (28.2-41.5)	0.732 (0.690-0.776)		0.568 (0.470-0.659)
kNN	Logarithm	1.48 (1.38-1.58)	1.34 (1.17-1.50)	32.7 (29.2-35.8)	33.3 (26.7-39.5)	0.569 (0.519-0.622)		0.433 (0.326-0.541)
Random forest regression	Logarithm	1.16 (1.08-1.25)	1.16 (1.01-1.31)	45.3 (41.7-48.7)	34.9 (28.2-41.5)	0.727 (0.685-0.774)		0.574 (0.487-0.665)
Gradient boosting	Logarithm	1.23 (1.15-1.32)	1.23 (1.07-1.38)	42.6 (39.0-46.0)	32.3 (25.6-39.0)	0.690 (0.644-0.740)		0.539 (0.435-0.641)
Ensemble model	Logarithm	1.12 (1.04-1.19)	1.14 (0.99-1.27)	47.6 (44.0-51.3)	33.8 (27.2-40.0)	0.750 (0.709-0.796)		0.578 (0.487-0.663)

^a^MARS: multivariate adaptive regression splines.

^b^kNN: k-nearest neighbors.

**Table 3:** Model performance for predicting the warfarin dose for a discharge international normalized ratio (INR) of 2.5-3.5 using validation data set.

Model	Outcome transformation	Mean absolute error (MAE), milligrams, 95% CI	Proportion of predictions within 20% of true dose (%), 95% CI	Correlation coefficient, 95% CI
Penalized Regression	None	1.17 (1.00-1.33)	41.2 (33.9-48.5)	0.502 (0.401-0.615)
MARS^a^	None	1.30 (1.13-1.47)	38.8 (30.9-46.1)	0.373 (0.228-0.554)
kNN^b^	None	1.26 (1.08-1.44)	38.2 (30.9-44.8)	0.339 (0.194-0.481)
Random Forest Regression	None	1.16 (0.99-1.33)	38.8 (31.5-46.7)	0.519 (0.408-0.642)
Gradient boosting	None	1.27 (1.07-1.46)	37.0 (29.7-44.2)	0.338 (0.209-0.469)
Ensemble Model	None	1.11 (0.94-1.27)	43.6 (35.8-50.9)	0.545 (0.442-0.660)
Penalized Regression	Logarithm	1.12 (0.94-1.29)	43.0 (35.2-50.3)	0.525 (0.419-0.641)
MARS	Logarithm	1.18 (0.99-1.35)	44.2 (35.8-51.5)	0.497 (0.372-0.650)
KNN	Logarithm	1.23 (1.03-1.42)	40.0 (32.1-47.3)	0.359 (0.244-0.472)
Random Forest Regression	Logarithm	1.19 (1.00-1.37)	42.4 (34.5-49.7)	0.464 (0.352-0.581)
Gradient boosting	Logarithm	1.30 (1.10-1.50)	38.2 (30.9-44.8)	0.361 (0.240-0.487)
Ensemble Model	Logarithm	1.16 (0.98-1.33)	41.2 (33.3-48.5)	0.507 (0.395-0.635)

^a^MARS: multivariate adaptive regression splines.

^b^kNN: k-nearest neighbors.

### Retrospective Case Series

**Figure 2:**
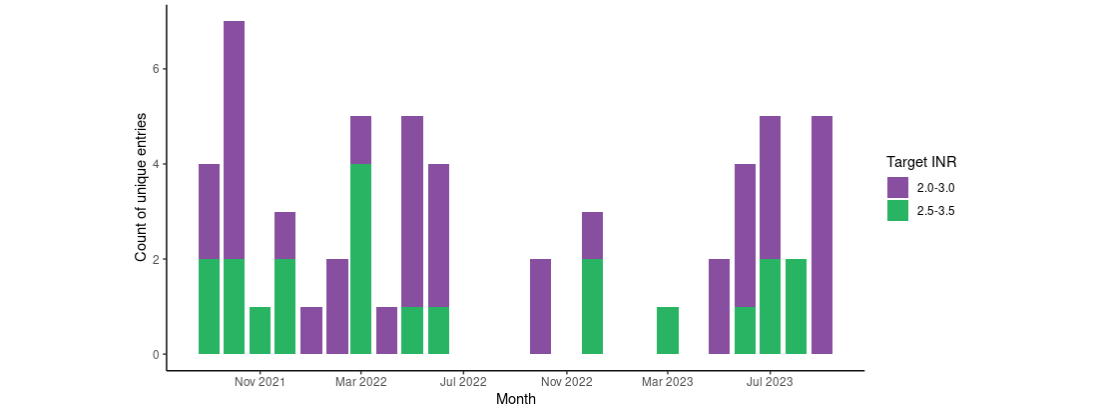
Warfarin dosing tool usage by St Michael’s Hospital cardiac surgery service, September 6, 2021, to September 18, 2023. INR: international normalized ratio.

The uptake of the warfarin dosing tool is illustrated in Figure 2.

**Figure 3:**
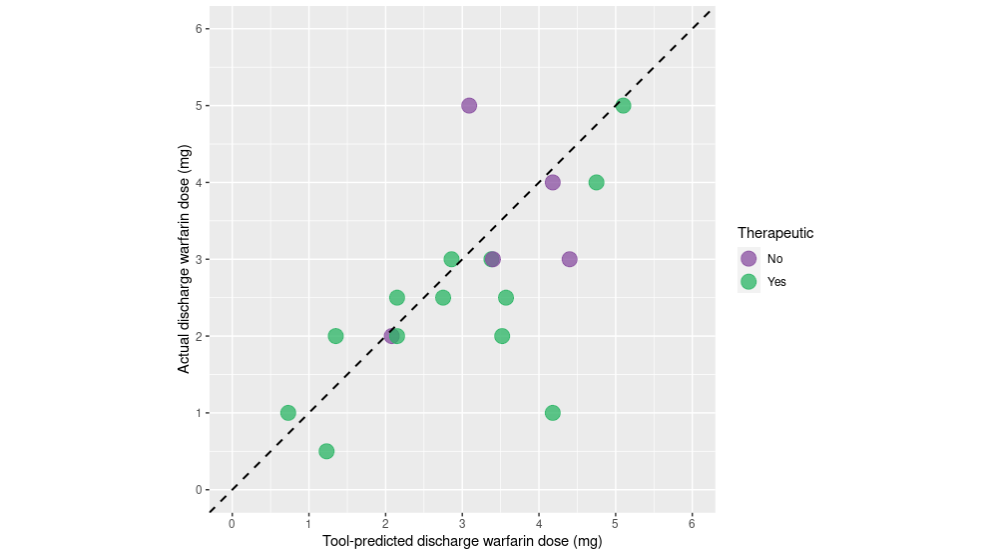
Predicted and true discharge warfarin doses for patients in the retrospective case series with indicators for whether their discharge international normalized ratio (INR) was therapeutic.

Because of COVID-19–associated reductions in surgical volumes at our institution, we identified only 18 warfarin-naïve patients who underwent cardiovascular surgery and were discharged on warfarin between September 2021 and May 2, 2022. Overall, 61.1% (11/18) of these patients were discharged with a therapeutic INR with a warfarin dose within the range predicted by the algorithm (Figure 3). In contrast, 47.5% (305/641) cardiovascular surgery patients were discharged with a therapeutic INR before the development and implementation of the web-based calculator (April 2011 to July 2019; *P*=.37 for pre- and postcomparison).

In a review of 7 patients with discrepancies between the predicted and actual discharge warfarin dose, a total of 2 patients were discharged with a therapeutic INR but with discharge doses lower than those predicted by the algorithm. Another 3 patients were not discharged with a therapeutic INR using the dose predicted by the algorithm. Of these patients, 2 were discharged with INRs that were nearly therapeutic (1.90 and 1.93; target INR 2.0-3.0), and 1 was discharged with an INR of 1.65 and therapeutic bridging with a low molecular weight heparin. The final 2 patients were not discharged with a therapeutic INR and were discharged with warfarin doses that differed from those predicted by the algorithm. A review of patient charts did not identify any factors that could account for discrepancies between actual and predicted discharge doses.

## Discussion

In this study, we found that machine learning models trained on readily available demographic and clinical data could predict the warfarin dose needed to discharge cardiac surgery patients with a therapeutic INR. Overall, random forest regression and ensemble models performed best for patients requiring discharge INRs of 2.0-3.0 and 2.5-3.5, respectively, with approximately 40% of predictions being within 20% of the actual dose. Based on our findings, a web-based tool was developed and deployed to the cardiovascular surgery unit of the hospital to facilitate warfarin dosing in the postoperative period. A preliminary evaluation found a numerically higher proportion of patients discharged with a therapeutic INR using the warfarin discharge dose predicted by the algorithm relative to historical controls. However, this evaluation was based on a small sample of patients, with additional evaluations being required.

Our findings are consistent with previous studies demonstrating the use of machine learning approaches for supporting personalized warfarin dosing in cardiac surgery patients. This is important because of the heightened sensitivity to warfarin in cardiac surgery patients during the postoperative period, thereby increasing the risk of bleeding and death or prolonging the length-of-stay because of delayed epicardial pacer wire removal [7,8,36,37]. Moreover, warfarin remains the only anticoagulant indicated for patients with mechanical mitral heart valves and remains commonly used following other forms of cardiac surgery [21-24,38]. In addition, our predictive algorithms were derived using clinical information that is routinely collected during the care of cardiac surgery patients. In contrast, some previous studies have integrated pharmacogenetic information accounting for up to 40% of individual variation in warfarin dosing with clinical data when developing and validating warfarin dosing algorithms [13,16,18,19,39]. Although a combination approach may improve model performance, these gains may be offset by the resources associated with obtaining pharmacogenetic data in all patients and the lack of availability of this information in most settings. Moreover, meta-analyses of randomized controlled trials comparing genotype-guided and clinical dosing algorithms have found inconsistent results with respect to time in the therapeutic range and bleeding risk, with no difference in mortality or thromboembolism risk [40,41]. However, the time to attain a therapeutic INR may be shortened by genotype-guided approaches [41]. Further, while cross-study comparisons are challenging, the MAEs for our best-performing models were similar to those of other studies incorporating pharmacogenetic data, with similar proportions of predictions falling within 20% of the true value. Specifically, the median MAEs of warfarin dosing algorithms were 1.20 (95% CI 0.37-3.70) [17] and 1.47-10.86 [13] in separate systematic reviews. Although this is higher than the MAE derived using a BPNN (0.740 mg) [14], our sample size limited us from exploring the performance of more advanced models. In addition, the median proportion of patients with the predicted dose within 20% of the true value was 48% from 14 studies describing the development of algorithms using clinical data only [17]. Our findings are also consistent with those of the Gage and International Warfarin Pharmacogenetics Consortium clinical algorithms, with a median and MAE of 1.50 and 1.41 mg/day, respectively [33,34].

Several limitations of this study merit emphasis. First, our sample size was small, potentially limiting the ability of models to recognize patterns and make predictions. However, this reflects our stringent inclusion criteria and emphasis on developing a warfarin dosing algorithm for a specific patient population. Moreover, we used expert knowledge and past research to select a parsimonious number of features for our predictive models. Further, our best-performing models had MAEs of approximately 1.1 mg, which is only slightly higher than the value of 1 mg considered to represent reasonable predictive ability [32]. Second, we lacked information on some variables known to affect warfarin sensitivity, such as smoking [42], ethnicity [43], and herbal medications [44]. Third, our case series examining the impact of our algorithms on clinical outcomes comprised a small number of patients because of COVID-19–associated reductions in surgical volumes. We were therefore likely underpowered to detect statistically significant differences in the proportion of patients discharged with a therapeutic INR following the implementation of the tool. Finally, we did not evaluate deep learning methods for algorithm development. However, we chose to avoid these methods because of the small number of observations in our data sets [14,15].

In conclusion, we found that a random forest regression and ensemble model based on routinely available clinical data provided accurate predictions to guide initial warfarin dosing in cardiac surgery patients requiring discharge INRs of 2.0-3.0 and 2.5-3.5, respectively. These algorithms can be harnessed to provide personalized warfarin dosing and optimize the postsurgical anticoagulation of these patients.

## Appendix

Multimedia Appendix 1 Additional information about each model.

## Abbreviations

BPNN: Back Propagation Neural Network
EDW: Enterprise Data Warehouse
INR: international normalized ratio
kNN: k-nearest neighbors
MAE: mean absolute error
MARS: multivariate adaptive regression splines
RR: responder ratio

## Data Availability

The data sets presented in this paper are not publicly available due to confidentiality restrictions preventing their distribution. Requests to access the data may be directed to the corresponding author.
