# Supplementary material for: Evaluation of Machine Learning Approaches for Predicting Warfarin Discharge Dose in Cardiac Surgery Patients: Retrospective Algorithm Development and Validation Study
Source: JMIR Cardio. 2023 Dec 6;7:e47262. doi: 10.2196/47262 (PMC10733832; doi:10.2196/47262)

**Supplemental Material: Additional information about each model**

Penalized linear regression is an extension of ordinary least squares that includes a regularization constraint in the model to shrink coefficient values towards zero relative to the least squares estimates. We used this approach to prevent model overfitting due to collinearity and high dimensionality, as well as to perform feature selection. The most commonly implemented penalty parameters are lasso (i.e., L1), ridge (i.e., L2) and elastic net regularization. Ridge regression adds the sum of the squares of coefficient estimates as a regularization term, while lasso regression adds the sum of the absolute values of the coefficient estimates as the penalty term to the loss function. Elastic net combines the two penalties as a weighted average of L1 and L2. The regularization term is multiplied by a hyperparameter called lambda to determine the amount of regularization added to the least squares loss function. For our study, we used elastic net regularization, identifying the optimal values of lambda and the proportion of L1 and L2 regularization in the model with grid search.

Random forest regression is an ensemble of independent decision trees created by using random bootstrap samples of the training observations and random subsets of the candidate variables in each split of the tree. The process begins by selecting a small random sample of explanatory features at the root node, and making the best split using this limited set of variables. A similar approach is applied for each subsequent node, with the entire process repeated with a new bootstrap sample. Because each tree is constructed using a random sample of the observations and features of the original dataset, random forest regression is less likely to overfit the training data than basic classification and regression tree models. The final prediction is obtained by calculating the mean of the predictions from the individual trees comprising the forest.

In contrast to random forest, which is an ensemble of independent trees, gradient boosting involves the stepwise construction of many small regression trees from the pseudo-residuals of previous trees. This sequential process combines the performance of weak regression trees to produce incremental improvements in model predictions.

MARS is a non-parametric modeling method that avoids the questionable linearity assumption of regular linear regression. Specifically, MARS approximates the nonlinear relationship between response and predictor variables by fitting the data into piecewise linear regression functions. Consequently, the slope of the regression function is permitted to change from one interval to the other. Moreover, MARS models evaluate interactions between variables and variable importance through a backwards elimination feature selection routine that determines the reduction in the generalized cross-validation estimate of error as each predictor is added to the model. As variables are automatically included or excluded during this process, MARS therefore also performs feature selection.

kNN regression is a nonparametric algorithm that makes predictions by averaging the outcomes of the observations most similar to the target (i.e., the k nearest neighbours), weighted by the inverse of their distance. We identified the optimal values of k (number of neighbours) and the weight function using grid search.

**Table S1.** Model hyperparameters.

| **Model Type** | **Hyperparameters** | **Hyperparameter Values/Ranges** |
| --- | --- | --- |
| Penalized Linear Regression | Penalty | 0.001-0.1 |
|  | Mixture | 0-1 |
| k-NN Regression | Number of Neighbours (k) | 1-15 |
|  | Weight Function | biweight, epanechnikov, rectangular, triangular, triweight |
| MARS | Interaction Degree | 1-2 |
|  | Number of Terms | 2-100 |
| Random Forest Regression | Number of Predictors to Sample at each Split (mtry) | 1-42 |
|  | Number of Observations Needed to Split (min_n) | 3-37 |
| Extreme Gradient Boosting (XGBoost) | Number of Observations Needed to Split (min_n) | 5-40 |
|  | Tree Depth | 2-14 |
|  | Learning Rate | 5.435168e-10 - 1.506040e-02 |
|  | Loss Reduction | 2.757167e-10 - 2.205715e+01 |

**Figure S1.** Predicted and true discharge warfarin doses for patients with a target international normalized ratio (INR) of 2.0-3.0, based on predictions generated on the test data set using the random forest regression model.


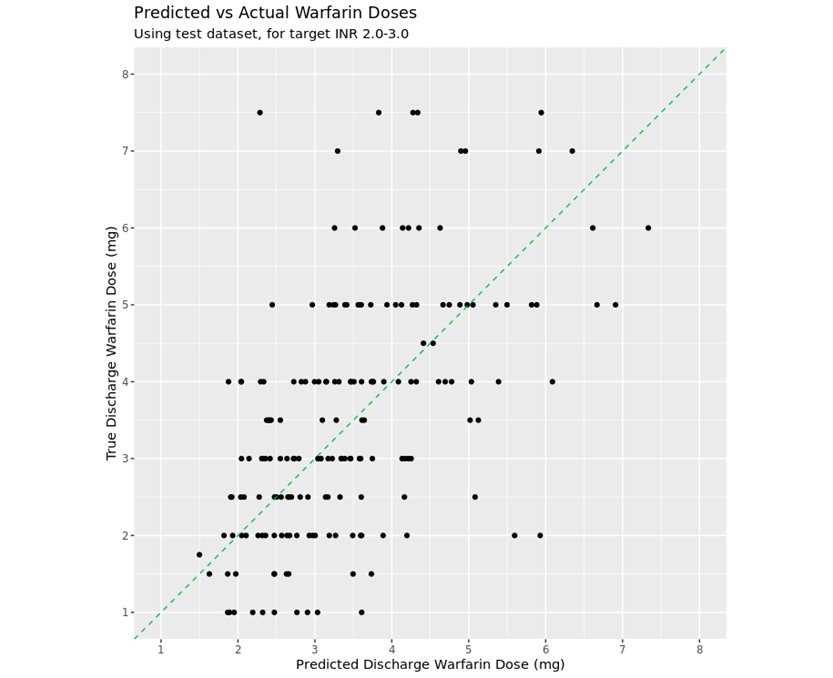


**Figure S2.** Predicted and true discharge warfarin doses for patients with a target international normalized ratio (INR) of 2.5-3.5, based on predictions generated on the validation dataset using the ensemble model.


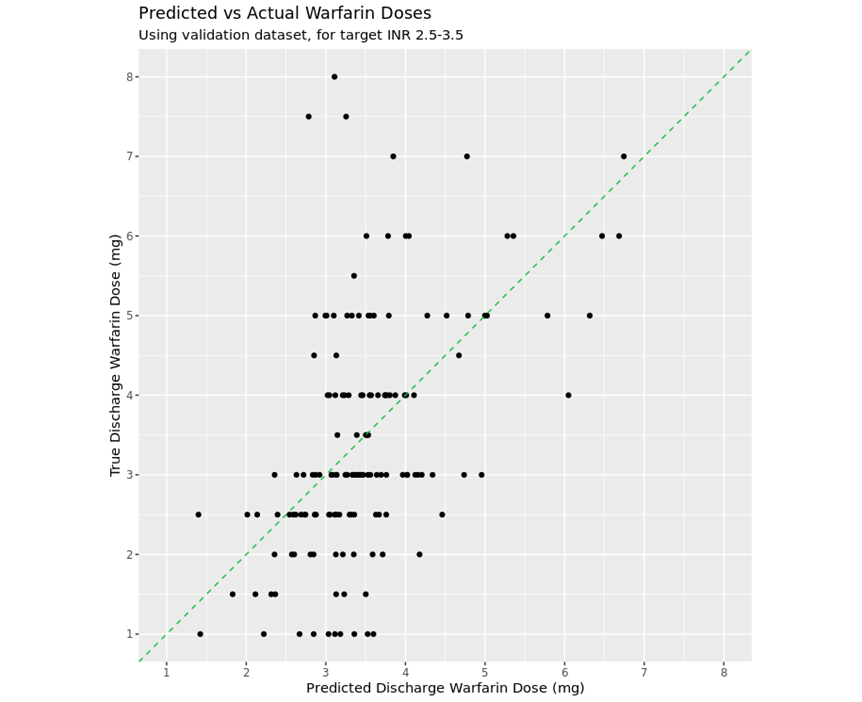

Supplement: Multimedia Appendix 1 [file cardio_v7i1e47262_app1.docx]
